# Functioning of women after mastectomy with subsequent breast reconstruction depending on cancer-related variables

**DOI:** 10.3389/fpubh.2026.1833840

**Published:** 2026-05-20

**Authors:** Ewelina Rogalska, Wiktoria Stenka, Ewa Kupcewicz, Marzanna Stanisławska, Daria Schneider-Matyka, Kamila Rachubińska, Elżbieta Grochans, Anna Maria Cybulska

**Affiliations:** 1 Independent Public Regional Combined Hospital in Szczecin, Szczecin, Poland; 2 Private Healthcare Facility, Maszewo, Poland; 3 Department of Nursing, Medical College, University of Warmia and Mazury, Olsztyn, Poland; 4 Department of Nursing, Faculty of Science, Pomeranian Medical University, Szczecin, Poland

**Keywords:** breast cancer, breast reconstruction, emotional control, mastectomy, psychological outcomes, self-efficacy, self-esteem, women

## Abstract

**Introduction:**

Breast cancer is the most common malignancy among women worldwide. Mastectomy, often accompanied by breast reconstruction, plays a key role in treatment and prevention among high-risk patients; however, psychological outcomes may vary depending on cancer-related factors.

**Aim:**

The aim of this study was to assess the relationship between selected cancer-related factors and psychological functioning in women after mastectomy with breast reconstruction.

**Method and material:**

This survey-based study involved 164 women after mastectomy with subsequent breast reconstruction. Psychological functioning was assessed using standardized psychometric instruments: the Satisfaction with Life Scale, Rosenberg Self-Esteem Scale, Generalized Self-Efficacy Scale, Courtauld Emotional Control Scale, and the Mini-Mental Adjustment to Cancer.

**Results:**

The study included 164 women who underwent mastectomy followed by breast reconstruction. The timing of the reconstruction decision wasn’t significantly associated with self-efficacy, self-esteem, life satisfaction, or overall psychological adjustment to cancer; however, women opting for immediate reconstruction demonstrated lower levels of emotional control, particularly in the anger domain, with a trend toward lower anxiety control. Longer disease duration was associated with reduced life satisfaction but more adaptive coping patterns, including greater positive reappraisal and lower helplessness. Participation in prehabilitation was associated with higher self-esteem only.

**Conclusion:**

The timing of the decision for breast reconstruction was not associated with self-efficacy, self-esteem, life satisfaction, or overall psychological adjustment. Immediate reconstruction after mastectomy was associated with less favorable emotional control indicators, although the practical significance of these effects appears limited. Longer illness duration was related to lower life satisfaction but more adaptive coping.

## Graphical abstract

**Figure:**
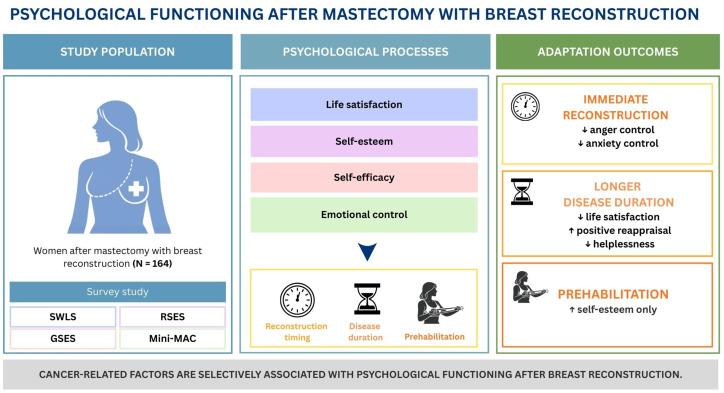

## 1 Introduction

Cancer is one of the leading causes of death worldwide. According to the World Health Organization (WHO), approximately one in six deaths globally in 2020 was attributable to cancer. Among women, breast cancer is the most frequently diagnosed malignancy and represents a major cause of cancer-related mortality, with an estimated 670,000 deaths worldwide in 2022. Epidemiological data show a steady increase in the global incidence of breast cancer, particularly in developed countries. Notably, more than half of diagnosed cases occur in the absence of identifiable risk factors other than age and sex. Breast cancer overwhelmingly affects women, accounting for approximately 99% of all cases (1). Documented and identified risk factors include excessive alcohol consumption, genetic factors, particularly BRCA1 and 2 gene mutations, obesity, smoking, age after 40, and the use of hormone replacement therapy. The course of the disease and prognosis are varied and largely dependent on the molecular subtype of the tumor, which determines the choice of therapeutic strategy (2, 3).

Breast cancer treatment includes hormone therapy, radiotherapy, and surgery. The gold standard surgical procedure is mastectomy, which is increasingly being performed as a preventive measure in patients at high risk of developing the disease. The decision to proceed with preventive treatment requires an individual analysis of the potential benefits and risks (4, 5). Mastectomy and cancer treatment are associated with numerous psychological consequences, including decreased self-esteem, body image disturbances, sexual problems, and an increased risk of depression and anxiety (6, 7). To reduce the severity and frequency of these complications, reconstructive procedures using implants or the patient’s own tissues are used. Reconstructions are performed concurrently with mastectomy or at a later date (8). Furthermore, comprehensive treatment, including post-rehabilitation, psychological support, and rehabilitation, is becoming increasingly popular in cancer treatment. Psycho-oncology plays a particularly important role, enabling patients to adapt more effectively to their illness, achieve faster mental recovery, and improve social functioning (9, 10).

Mastectomy, as a mutilating procedure, significantly affects body image, self-esteem, and the sense of femininity, as breasts are symbols of attractiveness and sexual identity, which may lead to stigmatization and the so-called “half-woman complex” (11). Women with breast cancer are at increased risk of depression, as well as anxiety and other mental health disorders (12). Risk factors include younger age, lack of social support, and lower socioeconomic status (13), while patients’ functioning depends on both internal and external factors. Sexual dysfunction is common among women after oncological treatment, and mastectomy is associated with a greater risk of such difficulties compared to breast-conserving surgery; moreover, a negative body image is linked to lower levels of sexual satisfaction (14). Breast reconstruction may reduce depressive symptoms, although its effect depends on the patient’s prior mental state, and severe major depression may constitute a contraindication to the procedure (12). Cancer diagnosis lowers quality of life, triggers strong negative emotions, and affects social and occupational functioning, while spirituality often serves a supportive role in coping with illness, although in some cases it may also intensify distress (15).

We focused on the analysis of medical data related to cancer and their relationship with the psychological functioning of patients. The study assessed the impact of prehabilitation, duration of cancer, and the timing of the decision to undergo breast reconstruction on the participants’ psychological state.

## 2 Materials and Methods

### 2.1 Background

A total of 174 women were included, of whom 164 completed the study. Patient enrollment was conducted through personal interviews. Patients ranged in age from 30 to 76 years, with a mean age of 49.8 ± 10.49 years. Participants were residents of the West Pomeranian Voivodeship. Each had undergone a mastectomy with subsequent breast reconstruction at the West Pomeranian Oncology Center in 2024 due to breast cancer.

Inclusion criteria included:

- no serious health problems unrelated to cancer in the study participants (e.g., mental illness requiring intensive treatment, chronic illness in the decompensation phase),
- no prior psychiatric treatment among the participants,
- confirmed by a clinical interview, age from 30 to 76 years,
- written informed consent to participate in the study and the processing of personal data,
- mastectomy and breast reconstruction performed at the West Pomeranian Oncology Center in 2024.

The exclusion criteria included the lack of informed consent of the woman to participate in the study, diagnosed mental illnesses occurring in the studied women (e.g., schizophrenia, severe depression, personality disorders), the presence of severe postoperative complications that prevented full recovery or required further surgical interventions, the presence of other types of cancer (not related to breast cancer), breast reconstruction not related to cancer as part of an esthetic medicine procedure, a previous mastectomy without breast reconstruction, serious health problems not related to cancer (e.g., chronic diseases in the decompensation phase), and incorrectly completed documentation. This publication forms part of a broader study examining the functioning of patients who underwent mastectomy for breast cancer followed by breast reconstruction. The findings presented here represent a subset of the overall results and focus specifically on the relationship between selected medical variables and the psychological functioning of women after mastectomy with subsequent breast reconstruction. The medical variables analyzed included the timing of the decision to undergo breast reconstruction, the duration of neoplastic disease, and patients’ use of prerehabilitation services.

Disease duration was operationalized as a dichotomous variable (<1 year vs. ≥1 year), reflecting a clinically meaningful distinction between the early phase of post-treatment adaptation and a more extended adjustment period. The first year following treatment is often considered a critical period of psychological and physical adaptation; therefore, this cut-off was used to differentiate between these stages.

### 2.2 Research tools and methods

The study employed a survey technique combined with a diagnostic survey. The survey included a proprietary questionnaire containing questions regarding sociodemographic data (gender, age, education, marital status, place of residence, professional activity) and cancer-related variables (duration of disease, decision to undergo breast reconstruction, pre-rehabilitation). Additionally, standardized research tools were used:

- The Generalized Self-Efficacy Scale (GSES) consists of 10 statements describing coping with difficult situations and obstacles. Perceived self-efficacy can be a determinant of health-related and other goals. The higher the self-efficacy, the higher the goals people set for themselves and the more committed they are to what they do, despite adversity. A total score of 10 to 40 is a general measure of self-efficacy. The higher the score, the greater the self-efficacy (Cronbach’s alpha = 0.84) (16).
- The Rosenberg Self-Esteem Scale (RSES) is a psychometric tool used to measure a participant’s self-esteem. The scale is a unidimensional tool that allows for the assessment of the level of general self-esteem—a relatively stable disposition understood as a conscious attitude (positive or negative) toward the self. The Rosenberg Self-Esteem Scale consists of 10 statements, each of which is diagnostic in nature. The participant is asked to rate the degree to which they agree with each statement, with responses provided on a four-point Likert scale. Scores on the SES range from 10 to 40 points, and higher scores indicate higher self-esteem (Cronbach’s alpha = 0.88) (17, 18).
- The Courtauld Emotional Control Scale (CECS) contains 21 items focusing on three basic emotions: anger, anxiety, and depression. The scale comprises three subscales, each containing seven items. The items used allow for the identification of forms of emotional suppression. This scale is intended for adults and allows for the assessment of subjective control of anger, anxiety, and depression. Respondents are asked to indicate the frequency of the suggested emotional expression on a scale: • “almost never” – 1, “sometimes” – 2, “often” – 3, and “almost always” – 4. The obtained scores are summed separately for each subscale, obtaining a score from 7 to 28 points. Then, adding the scores from all subscales yields a general emotional control index, which indicates the respondent’s subjective belief in their ability to control their emotions when experiencing negative emotions. The higher the score, the more effectively they suppress negative emotions (general score Cronbach’s alpha = 0.80) (19).
- The Satisfaction with Life Scale (SWLS) is a short instrument containing five items designed to subjectively assess and measure life satisfaction. The result is a general measure of life satisfaction. The scale is designed for adults, both healthy and sick. It is a useful tool for measuring life satisfaction (20). Scores are summed, and the overall score indicates the degree of satisfaction with one’s life. Higher scores indicate greater life satisfaction (Cronbach’s alpha = 0.81) (21).
- The Mini-MAC scale of psychological adjustment to cancer contains 29 items and measures four coping strategies. The Mini-MAC can be used to assess reactions to a cancer diagnosis and to capture changes occurring during treatment and rehabilitation. The scale’s scores can be an indicator of disease-related quality of life. The scale allows for estimating the frequency of coping strategies used. It addresses positive reappraisal and fighting spirit strategies, as well as the concepts of anxious preoccupation and helplessness. Higher scores indicate greater intensity of behaviors characteristic of a given coping style. Additionally, the Mini-MAC scale assesses two coping styles. Each style comprises two strategies: constructive style (strategies of fighting spirit and positive reappraisal), destructive style (strategies of anxious preoccupation and helplessness) (22). The higher the score, the greater the intensity of behaviors characteristic of a given way of coping with cancer (general score Cronbach’s alpha = 0.86) (23).

### 2.3 Sample size calculation

The sample size was determined pragmatically by the number of consecutive eligible women treated at the study center during the predefined recruitment period in 2024. No formal *a priori* sample size calculation was performed. As a sensitivity analysis, we estimated the minimum effect sizes detectable with the available sample under conventional assumptions (two-sided *α* = 0.05, power = 0.80). Given the observed group sizes, the study was powered primarily to detect moderate effects (approximately Cohen’s *f* = 0.25 for the three-group comparison and Cohen’s d = 0.44–0.46 for the main two-group comparisons), whereas smaller effects may have remained undetected.

### 2.4 Statistical analysis

Data were described using statistics appropriate to the level of measurement of the variables: for nominal variables, counts and percentages were reported; for ordinal variables, median, quartiles, and interquartile range (IQR); and for continuous variables on an interval/ratio scale, mean and standard deviation, plus median and quartiles. Where appropriate, 95% confidence intervals, reported as CI (Confidence Interval), were provided, including for the *ψ* estimate in pairwise comparisons.

Comparisons between groups were performed using robust one-way analysis of variance (robust ANOVA), appropriate for data potentially biased by non-normality and outliers; for factors with two levels, the Yuen test on trimmed means was used. Effect sizes (ES) and the *ξ* coefficient, along with their 95% CI, were reported. Robust ANOVA was performed using a bootstrap procedure with 9,999 resamples, the modified one-step estimator, and projection distances. *Post hoc* analyses used pairwise comparisons based on the *ψ* estimator, with 95% CIs enabling direct assessment of significance and direction of effects. Associations between quantitative/ordinal variables were assessed using the Spearman rank correlation coefficient (r), calculated for pairs of variables (e.g., age and psychological scales). Selected analyses were visualized using comparative boxplots presenting the median, quartiles, and outliers for each group.

For continuous outcome variables, multivariate models were constructed using classical least squares linear regression with psychometric predictors (e.g., GSES, SES, CECS, SWLS). Standardized *β* coefficients for each predictor and adjusted R^2^ for the entire model were reported. Analyses were conducted in Statistica version 13.3PL (TIBCO Inc.). All statistical tests were treated as two-sided, and the significance level was set at *α* = 0.05.

## 3 Results

### 3.1 Characteristics of the study sample

A total of 164 women aged 30–70 years who underwent mastectomy followed by breast reconstruction were surveyed. The mean age of the participants was 49.8 ± 10.49 years. The largest proportion of participants (39%) resided in cities with populations exceeding 100,000. Nearly half of the women had attained higher education (47.6%), and the majority were in formal relationships (64%). Most respondents were professionally active, including those employed under formal contracts (56.7%) and those who were self-employed (14%). Additionally, 65.9% of the participants reported having a good financial situation (Table 1).

**Table 1:** Characteristics of the study group, including variables related to mastectomy and breast reconstruction (*N* = 164).

%	n	Variables	
Duration of neoplastic disease	Less than a year	59	36.0
	More than a year	105	64.0
Timing of decision to undergo breast reconstruction	Before mastectomy (T0)	94	57.3
	Immediately after mastectomy (T1)	34	20.7
	One year or more after mastectomy (T2)	36	22,0
Motivation in making the decision about breast reconstruction	The desire to regain former appearance	99	60.4
	The desire to enhance self-esteem following the illness	54	32.9
	The desire to be perceived as attractive to one’s partner	13	7.9
	Other	18	11.0
Use of prerehabilitation	No	85	51.8
	Yes, dietitian	2	1.2
	Yes, physiotherapist	9	5.5
	Yes, psychologist	35	21.3
	Yes, psychologist/dietitian/physiotherapist	33	20.1

n, number of respondents.

Among the participants, 64% had a disease duration of 1 year or longer. Regarding the timing of the decision for breast reconstruction, 57.3% made the decision prior to mastectomy, 22% immediately after the procedure, and 20.7% 1 year or more following mastectomy. The most commonly reported motivation for undergoing breast reconstruction was the desire to regain one’s former appearance (60.4%). More than half of the respondents (51.8%) did not participate in post-rehabilitation. Among 21.3% received psychological support, while 20.1% benefited from multidisciplinary care involving a psychologist, dietitian, and physiotherapist (Table 1).

### 3.2 Timing of decision to undergo breast reconstruction

Data analysis revealed no statistically significant differences among participants with respect to self-efficacy, global self-esteem, or overall life satisfaction based on the timing of the decision to undergo breast reconstruction (Table 2). Comparisons between individual respondent groups, stratified according to the timing of this decision, demonstrated no statistically significant differences in the studied variables as assessed by the GSES, RSES, and SWLS instruments (Table 2).

**Table 2:** Self-efficacy according to GSES, self-assessment according to RSES, life satisfaction according to SWLS depending on the timing at which the decision to undergo breast reconstruction was made.

Variables	Decision regarding breast reconstruction			
	*F*	p*	Explained variance	*ES*
GSES	0.308	0.756	0.00955	0.0977
RSES	1.52	0.219	0.0355	0.188
SWLS	0.686	0.524	0.0229	0.151

*Analysis of variance (robust ANOVA); ES, effect size; *p*, significance level; GSES, generalized self-efficacy scale; RSES, Rosenberg Self-esteem scale; SWSL, Satisfaction with life scale; CECS, emotional control scale.

Data analysis revealed statistically significant differences in subjective overall emotion control according to the CECS depending on the timing of the decision to undergo breast reconstruction between the study groups, *F* = 3.98, *p* = 0.040. The explained variance for this model was 0.087, and the effect size (ES) was estimated at 0.294, indicating a moderate effect size. Similarly, significant differences were observed in subjective anger control according to the CECS, *F* = 7.14, *p* = 0.012. The explained variance for this model was 0.128, and the effect size (ES) was estimated at 0.358, indicating a moderate effect size. For the CECS anxiety subscale, the results approached statistical significance, *F* = 3.12, *p* = 0.053. The explained variance for this model was 0.0575, and the effect size (ES) was estimated at 0.240, indicating a moderate effect size. No statistically significant differences were observed on the CECS depression control subscale among respondents when stratified by the timing of the decision to undergo breast reconstruction (Table 3).

**Table 3:** Emotional control according to CECS depending on the timing at which the decision to undergo breast reconstruction was made.

Variables	Quality	Decision regarding breast reconstruction			
		F	p*	explained variance	ES
CECS	Overall control	3.98	**0.040**	0.0867	0.294
	Anger control	7.14	**0.012**	0.128	0.358
	Depression control	1.81	0.152	0.0566	0.216
	Anxiety result	2.12	**0.053**	0.05750	0.240

*analysis of variance (robust ANOVA); ES, effect size; *p*, level of significance; CECS, Emotional Control Scale. Bold values indicate statistically significant results (*p* < 0.05).

*Post hoc* tests analyzing differences between groups: before surgery (T0), immediately after surgery (T1), and 1 year or more after surgery (T2), revealed partially significant differences in CECS scores. The comparison between the T0 and T1 groups yielded a *ψ* estimate of −11.26 (*p* = 0.010) with a 95% confidence interval of −19.62 to −1.87, indicating a significant difference. Women who opted for breast reconstruction immediately after mastectomy demonstrated the lowest levels of emotional control according to the CECS. Differences between the other groups were not statistically significant. For the anger domain of the CECS, comparing the pre-treatment group (T0) with the immediate post-treatment group (T1) revealed a difference estimate of *ψ* of −4.90 (*p* = 0.003) with a 95% confidence interval of −7.544 to −1.890. Comparing the T1 and T2 groups yielded a difference estimate of ψ = 3.318 (*p* = 0.039); however, the corresponding 95% confidence interval included zero (−0.409 to 6.591), and this contrast should therefore be interpreted cautiously. Comparing the T0 group with the T2 group revealed no significant difference in anger control. There were no significant differences between the groups in terms of depression control on the CECS scale. For the CECS anxiety subscale, the omnibus model did not reach formal statistical significance (*p* = 0.053). Nevertheless, an exploratory pairwise comparison suggested lower anxiety control in the T1 group compared with the T0 group (*ψ* = −3.87, *p* = 0.010; 95% CI, −7.09 to −0.20), and this finding should be interpreted cautiously. No statistically significant differences were found between the other groups in the anxiety domain of the CECS scale. (Table 4).

**Table 4:** *Post-hoc test*—control of emotions according to CECS depending on the time of making the decision about breast reconstruction.

CECS	Decision regarding breast reconstruction				95% confidence interval	
	Pairwise comparison		Psi*	*p*	Lower	Upper
Overall result	Before (T0)	Right after (T1)	−11.260	**0.010**	−19.620	−1.870
	Before (T0)	Year after (T2)	−3.940	0.240	−11.860	3.820
	Right after	Year after (T2)	7.320	0.125	−3.270	15.590
Anger control	Before (T0)	Right after (T1)	−4.900	**0.003**	−7.544	−1.890
	Before (T0)	Year after (T2)	−1.580	0.200	−4.342	1.090
	Right after (T1)	Year after (T2)	3.318	**0.039**	−0.409	6.591
Depression control	Before (T0)	Right after (T1)	−2.610	0.067	−5.660	0.903
	Before (T0)	Year after (T2)	−0.0.610	0.611	−3.470	2.346
	Right after (T1)	Year after (T2)	2.000	0.209	−1.680	5.636
Anxiety control	Before (T0)	Right after (T1)	−3.870	**0.010**	−7.090	−0.204
	Before (T0)	Year after (T2)	−1.550	0.307	−4.360	2.132
	Right after (T1)	Year after (T2)	2.320	0.175	−1.270	6.182

Estimator of the difference ψ; *p*, level of significance; CECS, Emotional Control Scale. Bold values indicate statistically significant results (*p* < 0.05).

While this interpretation suggests that the timing of reconstruction may be associated with differences in emotional functioning, caution is warranted. An equally plausible alternative explanation is that women with lower pre-existing emotional regulation capacity were more likely to choose immediate reconstruction as a more reactive response to distress, rather than as a decision made from a position of psychological stability.

Data analysis revealed no statistically significant differences in the level of psychological adaptation to cancer, as measured by the Mini-MAC scale, among the women studied according to the timing of the decision to undergo breast reconstruction. Analysis of variance likewise showed no statistically significant differences between groups with respect to specific coping styles, including anxious preoccupation, fighting spirit, helplessness–hopelessness, and positive reappraisal (Table 5). *Post-hoc* analyses further confirmed the absence of statistically significant differences among women who decided on breast reconstruction before mastectomy, immediately after the procedure, or 1 year or more thereafter.

**Table 5:** Psychological adaptation to cancer according to the Mini-MAC scale of the surveyed women depending on the time of making the decision about breast reconstruction.

Mini-MAC	F	Decision regarding breast reconstruction		
		p*	explained variance	ES
Anxious preoccupation	0.348	0.696	0.00865	0.093
Fighting spirit	0.0964	0.903	0.00432	0.0657
Helplessness-hopelessness	0.192	0.840	0.00436	0.0066
Positive redefinition	1.68	0.199	0.0354	0.188
Constructive style	0.793	0.462	0.015	0.122
Destructive style	0.608	0.584	0.00797	0.0893

*analysis of variance (robust ANOVA); ES, effect size; *p*, level of significance; Mini-MAC, scale of mental adjustment to cancer.

### 3.3 Duration of neoplastic disease

Comparative analyses between patients with an illness duration of 1 year or longer and those with a duration of less than 1 year revealed no statistically significant differences in self-efficacy according to the GSES, self-esteem according to the RSES, or emotional control as measured by the CECS across its domains (anger, depression, anxiety, and total score). In contrast, statistically significant differences were observed in life satisfaction (SWLS), with patients experiencing illness for 1 year or longer reporting lower perceived life satisfaction than those with a shorter illness duration. The observed effect size (*ξ* = 0.351; 95% CI: 0.100–0.581) indicates a moderate effect, consistent with established interpretations in psychological and social science research (Table 6). Illness duration was also identified as a significant factor influencing selected domains of psychological adaptation to cancer, as assessed by the Mini-MAC scale. Patients with an illness duration of 1 year or longer demonstrated higher levels of positive reappraisal and lower levels of anxious preoccupation as well as helplessness–hopelessness compared with those with a shorter illness duration. No statistically significant differences were found between groups in the fighting spirit domain. The largest effect size was observed for helplessness–hopelessness (*ξ* = 0.461), indicating the most pronounced between-group differences, followed by positive reappraisal (ξ = 0.429). The smallest, though still moderate and statistically significant, effect was noted for anxious preoccupation (ξ = 0.382; Table 6). Additionally, significant between-group differences were identified in the severity of constructive and destructive coping styles on the Mini-MAC scale after accounting for illness duration.

**Table 6:** Self-efficacy according to GSES, self-esteem according to SES, life satisfaction according to SWLS, emotional control according to CECS, psychological adaptation to the disease according to Mini-MAC depending on the duration of the cancer disease.

Variable	Quality					95% confidence interval	
		t	df	p*	ξ**	Lower	Upper
GSES		1.194	74.7	0.236	0.144	0.000	0.394
CECS	Overall control	0.173	67.3	0.863	0.042	0.000	0.298
	Anger control	0.874	65.3	0.385	0.102	0.000	0.378
	Depression control	0.181	67.9	0.857	0.047	0.000	0.312
	Anxiety result	0.451	61.7	0.654	0.065	0.000	0.331
SWLS		2.973	82.5	**0.004**	0.351	0.100	0.581
Mini-MAC	Anxious preoccupation	3.827	77.8	**<0.001**	0.382	0.151	0.608
	Fighting spirit	1.731	95.3	0.087	0.202	0.000	0.461
	Helplessness-hopelessness	3.916	84.8	**<0.001**	0.461	0.196	0.657
	Positive redefinition	3.888	91.3	**<0.001**	0.429	0.188	0.631
	Constructive style	3.045	75.4	**0.003**	0.367	0.130	0.585
	Destructive style	3.811	84.8	**<0.001**	0.408	0.175	0.624
RSES		0.285	92.6	0.776	0.061	0.000	0.391

*Yuen’s test, **effect size (coefficient, GSES, Generalized Self-Efficacy Scale; CECS, Emotional Control Scale; SWSL, Satisfaction with Life Scale; Mini-MAC, Scale of Mental Adjustment to Cancer; SES, Self-Assessment Scale). Bold values indicate statistically significant results (*p* < 0.05).

Patients with an illness duration of less than 1 year exhibited higher levels of destructive coping styles, which may reflect greater difficulties in psychological adaptation during the early stages of the disease. In contrast, patients with an illness duration exceeding 1 year demonstrated higher levels of constructive coping styles compared with those with a shorter illness duration. The effect size for destructive coping styles was *ξ* = 0.4084 (95% CI: 0.175–0.624), indicating a moderate-to-large effect, whereas the effect size for constructive coping styles was *ξ* = 0.3673 (95% CI: 0.130–0.585), reflecting a moderate effect (Table 6).

### 3.4 The use of prehabilitation

Data analysis revealed statistically significant differences in overall self-esteem, as measured by the SES scale, between patients who participated in prehabilitation and those who did not. Participants who underwent prehabilitation reported higher self-esteem than non-participants, with a moderate effect size (ξ = 0.220; 95% CI: 0.025–0.552; Table 7). In contrast, no statistically significant between-group differences were observed for self-efficacy (GSES), life satisfaction (SWLS), emotional control (CECS), or psychological adaptation to cancer (Mini-MAC; Table 7).

**Table 7:** Self-efficacy according to GSES, self-esteem according to SES, life satisfaction according to SWLS, emotional control according to CECS, psychological adaptation to the disease according to Mini-MAC depending on the use of prehabilitation.

Variable	Quality					95% confidence interval	
		t	df	p*	ξ**	Lower	Upper
GSES		0.045	94.3	0.965	0.016	0.000	0.261
CECS	Overall control	0.187	95.0	0.852	0.023	0.000	0.259
	Anger control	0.551	96.6	0.583	0.070	0.000	0.293
	Depression control	0.087	96.7	0.931	0.019	0.000	0.254
	Anxiety result	0.040	96.7	0.969	0.017	0.000	0.260
SWLS		0.096	97.5	0.924	0.015	0.000	0.245
Mini-MAC	Anxious preoccupation	1.726	95.4	0.088	0.182	0.000	0.377
	Fighting spirit	−0.591	80.0	0.556	0.072	0.000	0.290
	Helplessness-hopelessness	0.860	98.0	0.392	0.101	0.000	0.308
	Positive redefinition	0.436	97.0	0.664	0.046	0.000	0.244
	Constructive style	0.086	94.4	0.932	0.019	0.000	0.262
	Destructive style	1.327	97.7	0.188	0.140	0.000	0.352
RSES		2.332	98.0	**0.022**	0.220	0.025	0.552

*Yuen’s test, ** effect size (ksi coefficient), GSES, Generalized Self-Efficacy Scale; CECS, Emotional Control Scale; SWSL, Satisfaction with Life Scale; Mini-MAC, Scale of Mental Adjustment to Cancer; SES, Self-Assessment Scale. Bold values indicate statistically significant results (*p* < 0.05).

## 4 Discussion

Breast cancer represents one of the most significant challenges in contemporary medicine and public health. It is the most frequently diagnosed malignant neoplasm among women worldwide, and its incidence continues to increase. Despite advances in diagnostics and treatment, breast cancer remains one of the leading causes of cancer-related mortality among women. Surgical treatment constitutes the cornerstone of breast cancer therapy (1, 24). Breast reconstruction, in turn, is an important component of the recovery process following mastectomy and has a substantial impact on patients’ quality of life and psychosocial functioning (25). Although not all patients are eligible for immediate reconstruction, available evidence indicates that breast reconstruction provides quality-of-life benefits even among women with a poor oncological prognosis (26). Furthermore, studies demonstrate that reconstruction is associated with better disease adaptation, lower levels of anxiety, and reduced tendencies toward depression and addictive behaviors (27).

### 4.1 Timing of the decision to undergo reconstruction and psychological functioning

The present study examined associations between selected cancer-related variables and the psychological functioning of women after mastectomy and subsequent breast reconstruction. Three factors were analyzed: the timing of the decision to undergo reconstruction, disease duration, and participation in prehabilitation.

The findings indicate that the timing of the decision to undergo breast reconstruction was not significantly associated with self-efficacy, global self-esteem, or life satisfaction. These results are partially consistent with the study by Pačarić et al. (28), which included 79 patients divided into three groups: women who declined reconstruction, those who underwent immediate reconstruction, and those who underwent delayed reconstruction. Although the group that declined reconstruction demonstrated significantly lower quality of life, no clear differences were observed between the immediate and delayed reconstruction groups depending on the timing of the procedure. This suggests that the fact of undergoing reconstruction may be more important for quality of life than the timing of the decision itself.

Similarly, no significant associations were found between the timing of the reconstruction decision and psychological adjustment to cancer, as measured by the Mini-MAC scale. Coping styles including fighting spirit, helplessness–hopelessness, anxious preoccupation, and positive reappraisal did not differ depending on when the decision was made. These findings support the hypothesis that coping strategies are more strongly determined by relatively stable personality traits as well as social and environmental factors than by a single therapeutic decision (29, 30).

This interpretation is further supported by research indicating that personality traits influence the willingness to undergo reconstruction. Bochtsou et al. (31) demonstrated that older age and lower openness to experience were associated with reduced willingness to pursue reconstruction and decreased interest in seeking information about the procedure. Lower readiness for reconstruction was also observed among women with poorer physical quality of life and among patients with higher levels of depressive symptoms. These findings suggest that the decision-making process is complex and influenced by both personality characteristics and current psychophysical condition.

### 4.2 Emotion regulation and timing of decision

Significant differences were observed in overall emotion control and anger regulation, with a trend toward differences in anxiety. Women who made the decision to undergo reconstruction in the immediate postoperative period demonstrated lower levels of emotional regulation compared to those who decided before mastectomy or at a later stage. Although some associations reached statistical significance, the proportion of explained variance was small to modest, indicating that the timing of the reconstruction decision accounted for only a limited part of the variability in emotional control outcomes. Therefore, these findings should be interpreted as suggestive rather than definitive from a clinical perspective, and they do not support strong clinical recommendations in isolation.

This may indicate that the immediate postoperative period coincides with increased emotional vulnerability, psychological burden, and diminished regulatory capacity. The perioperative period is typically characterized by acute stress, uncertainty, and physical discomfort, all of which may impair adaptive emotional processes. However, findings in the literature are not unequivocal. Some studies report positive psychological outcomes associated with immediate reconstruction (32), whereas others emphasize the negative impact of prolonged waiting times, which have been linked to lower quality of life (33).

Longitudinal studies further suggest that differences between immediate and delayed reconstruction may be temporary. Zhong et al. (34) found that although groups initially differed in body image, sexual satisfaction, and psychosocial functioning, these differences were no longer statistically significant 1–1.5 years after reconstruction. Similarly, Yoon et al. (35), in a large cohort of nearly 2,000 patients, reported initial advantages of immediate reconstruction in terms of breast satisfaction, mental health, and sexual functioning; however, these differences diminished over a two-year follow-up period.

A systematic review by Roy et al. (7) also highlighted the heterogeneity of findings, with some studies demonstrating psychological benefits of immediate reconstruction and others reporting no significant long-term differences.

Collectively, these findings suggest that immediate reconstruction may shorten the duration of transitional adjustment difficulties, although long-term psychosocial outcomes appear comparable between surgical approaches. These findings support an individualized approach to clinical decision-making and highlight the importance of considering patients’ psychological resources, although the practical significance of the observed effects appears limited.

### 4.3 Disease duration and psychological adaptation

Disease duration did not significantly differentiate overall emotional regulation; however, it emerged as a significant predictor of life satisfaction. Women who had been diagnosed for more than 1 year reported lower life satisfaction compared to those with a shorter disease duration. This may reflect the cumulative burden of prolonged treatment and chronic health-related stress. Conversely, longer disease duration was associated with better psychological adjustment to cancer, including higher levels of positive reappraisal and lower levels of helplessness and anxious preoccupation. These findings suggest a gradual process of psychological adaptation, in which patients develop more constructive coping strategies over time.

Similar conclusions were drawn by Metcalfe et al. (36), who observed improvements in psychosocial well-being over time following mastectomy. Moreover, Hsu et al. (37) demonstrated that, in long-term follow-up, women with a history of breast cancer did not significantly differ in quality of life from women without a cancer history. These results highlight the importance of providing psychological support particularly in the early post-diagnosis period, which appears to be the most emotionally demanding stage.

### 4.4 The role of prehabilitation

Prehabilitation was significantly associated with higher self-esteem, although no differences were observed in self-efficacy, life satisfaction, emotional control, or psychological adjustment to cancer. It is possible that supportive interventions enhance patients’ sense of agency and empowerment, thereby directly influencing self-evaluation.

A systematic review by Toohey et al. (38), encompassing 14 studies, demonstrated that prehabilitation programs including physiotherapy, psychological support, smoking cessation, and multimodal interventions were associated with improved physical functioning and lower levels of anxiety and depressive symptoms. Additionally, patients express strong interest in participating in such programs (39), supporting the need for further research on their impact on both clinical and psychosocial outcomes.

The findings of the present study suggest that the timing of the decision to undergo breast reconstruction is not a primary determinant of long-term psychological functioning, although it may influence short-term emotional regulation. Disease duration appears to reflect a dynamic process of psychological adaptation, with the period immediately following diagnosis representing a particularly burdensome and vulnerable stage. Collectively, these results underscore the importance of individualized decision-making regarding the timing of reconstruction, with careful consideration of patients’ personality traits and psychological resources. They also support careful psychological assessment and a personalized clinical approach, while avoiding strong causal or practice recommendations based on the present findings alone. Further research is needed to clarify the psychosocial effects and clinical relevance of prehabilitation.

### 4.5 Limitations

A strength of this study is the inclusion of 164 patients and the comprehensive assessment of psychological functioning. Nevertheless, several methodological limitations should be acknowledged. First, the study was geographically restricted to a single voivodeship within one country, limiting the ability to account for sociocultural and healthcare system differences that may influence psychological adaptation. Moreover, all oncological and surgical treatments were conducted at a single medical center. While this ensured procedural consistency, the use of a uniform therapeutic approach and medical team may limit the generalizability of the findings to other clinical settings. Second, the relatively narrow time frame of data collection restricted to patients treated in 2024 may constrain the applicability of the results in the context of rapidly evolving oncological, reconstructive, and psychological care standards. In addition, the study did not incorporate a detailed analysis of key clinical and contextual variables that may influence psychological outcomes following mastectomy, including cancer stage, type of reconstruction (implant-based vs. autologous), receipt of adjuvant chemotherapy or radiotherapy, time elapsed since surgery at the time of psychological assessment, menopausal status, level of social support, and patients’ physical and functional status. Another important limitation is the cross-sectional design, which precludes conclusions about causal relationships. In particular, it remains unclear whether the timing of breast reconstruction influences psychological functioning, or whether pre-existing emotional characteristics influenced the timing of the decision. Furthermore, prehabilitation was operationalized as a binary variable (yes/no), despite encompassing heterogeneous forms of support such as physiotherapy, psychological interventions, multimodal care, and dietary counseling. These modalities differ substantially in their nature and mechanisms of action. Due to the small sample sizes within individual subgroups, separate analyses were not feasible; however, this aggregation may have obscured meaningful differences and should be considered when interpreting the results, particularly those presented in Section 3.4.

Because no formal *a priori* sample size calculation was performed and some subgroup and pairwise comparisons involved relatively small sample sizes, the study may have been underpowered to detect small effects. Therefore, non-significant findings should be interpreted cautiously, particularly in the smaller subgroup analyses.

Finally, the reliance on self-report instruments, although based on validated psychometric tools, introduces the possibility of bias related to subjective perception, current emotional state, and socially desirable responding. Taken together, these limitations highlight the need for future research incorporating more diverse populations, longitudinal designs, more granular clinical data, and multicenter approaches to enhance the robustness and generalizability of findings.

## 5 Conclusion

- The timing of the decision to undergo breast reconstruction was not associated with self-efficacy, self-esteem, life satisfaction, or overall psychological adjustment to cancer.
- Patients who decided on breast reconstruction immediately after mastectomy showed lower anger control, with a tendency toward lower anxiety control; however, these associations explained only a limited proportion of outcome variability and should be interpreted cautiously.
- Longer illness duration was associated with reduced life satisfaction but more adaptive coping, including greater positive reappraisal and lower anxious preoccupation and helplessness.
- Participation in prehabilitation was associated with higher self-esteem, with no significant effects observed for other psychological outcomes.
- Further studies with larger samples are needed to confirm these findings and to identify additional factors influencing psychological functioning after mastectomy and breast reconstruction.

## Data Availability

The raw data supporting the conclusions of this article will be made available by the authors, without undue reservation.
